# Supraphysiological estradiol promotes human T follicular helper cell differentiation and favours humoural immunity during in vitro fertilization

**DOI:** 10.1111/jcmm.16651

**Published:** 2021-05-24

**Authors:** Cong Hu, HongLei Liu, Bo Pang, Hao Wu, Xiuying Lin, Yu Zhen, Huanfa Yi

**Affiliations:** ^1^ Central Laboratory The First Hospital of Jilin University Changchun China; ^2^ Center for Reproductive Medicine Center for Prenatal Diagnosis The First Hospital of Jilin University Changchun China; ^3^ Department of Rheumatology and Immunology Shanghai Jiao Tong University School of Medicine Affiliated Ruijin Hospital Shanghai China; ^4^ Department of Cardiology The First Hospital of Jilin University Changchun China; ^5^ Department of Nephrology The First Hospital of Jilin University Changchun China; ^6^ Center for Reproductive Medicine Jilin Province People's Hospital Changchun China; ^7^ Department of Dermatology and Venerology The First Hospital of Jilin University Changchun China; ^8^ Key Laboratory of Organ Regeneration and Transplantation Ministry of Education Changchun China

**Keywords:** B cells, estradiol, humoural immunity, in vitro fertilization, T follicular helper cells

## Abstract

During pregnancy, humoural immunity is essential for protection against many extracellular pathogens; however, autoimmune diseases may be induced or aggravated. T follicular helper (Tfh) cells contribute to humoural immunity. The aim of this study was to test whether Tfh cell function can be manipulated via hormones. Seventy‐four women who underwent in vitro fertilization were recruited and divided into four groups: menstrual period (MP), controlled ovarian hyperstimulation (COH), embryo transfer (ET) and pregnant after embryo transfer (P). A flow cytometry analysis was performed to identify Tfh cells in peripheral blood mononuclear cells (PBMCs). Bioinformatics analysis revealed a possible pathway between Tfh and B cells. Enzyme‐linked immunosorbent assays were used to detect interleukin (IL)‐21 and IL‐6. The quantitative polymerase chain reaction was performed to quantify BCL‐6, BACH2, XBP‐1, IRF‐4 and G protein‐coupled (GP)ER‐1 mRNA expression. Compared with the MP group, the COH, ET and P groups showed more Tfh and B cells, as well as higher IL‐21, IL‐6, BCL‐6 and BACH2 expression. Furthermore, Tfh cell frequency in PBMCs, as well as serum IL‐21 and IL‐6 levels, were all positively correlated with serum estradiol (E_2_) levels; the B cell percentage also correlated positively with Tfh cells in PBMCs. Combined with the bioinformatics analysis, XBP‐1, IRF‐4 and GPER‐1 expression was related to E_2_ levels, both in vivo and in vitro. We speculate that E_2_ augments Tfh cells and favours humoural immunity. This study indicates that Tfh cell regulation may be a novel target in maintaining the maternal‐foetal immune balance.

## 1 INTRODUCTION

Pregnancy is characterized by high levels of both oestrogen and progesterone in vivo.^1^, ^2^, ^3^ Changes in these hormones during pregnancy appear to inhibit the cellular immune response classically mediated by T helper (Th)1/Th2 and Th17/T regulatory cells^4^, ^5^ and, in contrast, increase antibody production by memory B lymphocytes.^6^ It has been reported that in some Th1‐driven autoimmune diseases, pregnancy can temporarily abate the clinical signs in the mother.^7^, ^8^ Humoural immunity is essential for protection against extracellular pathogens; however, excess autoantibody production during pregnancy may induce or aggravate autoimmune diseases such as rheumatic antiphospholipid syndrome and systemic lupus erythematosus.^9^, ^10^, ^11^ The mechanism underlying the relationship between maternal hormones and humoural immunity remains unclear.

T follicular helper (Tfh) cells are a unique subset of CD4^+^ T cells that play important roles in germinal centre (GC) formation and high‐affinity antibody generation.^12^, ^13^ Tfh cells and GC B cells share some transcriptional networks, and many molecular pathways regulating Tfh cells can be deduced from B cells.^14^ Tfh cells, first observed in human lymphoid tissues, are characterized by IL‐21 and IL‐6 release and the expression of some unique molecules and factors, such as CD4, B cell lymphoma 6 (BCL6), inducible co‐stimulatory molecule (ICOS), CXC chemokine receptor 5 (CXCR5) and programmed death‐1 (PD‐1). Thus, Tfh cells are defined as CD4^+^BCL6^+^ICOS^+^CXCR5^+^ PD‐1^+^.^13^ BCL6 is the master transcriptional factor for the development of both GC B cells and Tfh cells induced by IL‐6,^15^, ^16^, ^17^ whereas CXCR5 is induced by IL‐21 and ICOS.^18^, ^19^ In addition, BCL6 is necessary for early CXCR5 expression.^20^, ^21^, ^22^

As a subset that facilitates T cell‐dependent B cell activation, Tfh cells play important roles in autoreactive B cell proliferation, promoting the production of autoantibodies.^23^ During pregnancy, Tfh cell numbers increase in both mice and human beings and decrease in case of a miscarriage.^24^, ^25^ When PD‐1 or IL‐21 are blocked, spontaneous abortion is high in mice as a consequence.^25^, ^26^

Estradiol (E_2_) is the predominant hormone in females that plays pivotal roles in endocrine and reproductive systems. E_2_ also manipulates immune cells in autoimmune diseases and exacerbates some cancers.^27^, ^28^ In mice, E_2_ treatment decreases the Tfh cell response and suppresses *Bcl6* and *Il‐21* mRNA expression to control autoantibody production;^29^ however, the effect of E_2_ on Tfh cells in humans is still obscure. Controlled ovarian hyperstimulation (COH) with supraphysiological E_2_ and without a progesterone antagonist is an approach to assist in vitro fertilization (IVF). It can be used to explore the relationship between E_2_ and Tfh cells, B cells and immunity in human beings.

In this study, we aimed to observe and identify the relationship between Tfh and B cell numbers, and E_2_ levels, during IVF. We also determined the levels of IL‐6, IL‐21, BCL6 and BACH2, and their correlation with E_2_ levels.

## 2 MATERIAL AND METHODS

### 2.1 Patients’ samples

Women of an average age of 30.23 ± 3.06 years, undergoing IVF treatment (n = 74), were recruited from March to October 2019 at the Reproductive Medicine Center of Jilin Province People's Hospital (Supplementary Table S1). The inclusion criteria were as follows: (i) first IVF cycle, (ii) less than 35 years old, (iii) good ovarian response, (iv) normal karyotype and hormones and (v) without uterine abnormalities (fibroid, uterine septum, and uterine polyp), endometriosis or any other known immunological diseases.

Luteal‐phase GnRH‐a protocol was performed in all patients. GnRH‐a (Triptorelin Acetate Injection) was used to suppress pituitary, then, recombinant follicle‐stimulating hormone (Gonal‐F) and human menopausal gonadotropin (menotrophins for injection) were used for ovarian stimulation. When the dominant follicles (three or more) reached 18 mm in diameter, human chorionic gonadotrophin for injection (HCG) was administered. Transvaginal ultrasound‐guided oocyte retrieval was performed 36 hours later. Luteal phase support was performed using 90 mg of vaginal progesterone (Crinone gel 8%), administered from the oocyte retrieval day. Three‐day later, two high‐quality embryos were transferred into an adequately prepared endometrium. Early pregnancy was defined as the presence of an intrauterine gestational sac, with positive β‐hCG results 4 weeks later. Luteal phase support was continued until 9 weeks gestation (almost 7 weeks after embryo transfer).

Patients were divided into four groups according to the stage of IVF treatment, menstrual period (MP), n = 20; controlled ovarian hyperstimulation (COH), n = 25; embryo transfer (ET), n = 15; and pregnant after embryo transfer (P), n = 14. The MP group was the basic hormone group with low E_2_ and progesterone; the COH group had hyperphysiological E_2_ levels without progesterone; the ET group had high E_2_ and progesterone levels (progesterone is an antagonist of E2); and the P group had higher E_2_ and progesterone levels.

### 2.2 Blood mononuclear cells isolation and flow cytometry

Blood from peripheral and cord vein was collected in sterile heparinized tubes from each patient. Peripheral blood mononuclear cells (PBMC) and cord blood mononuclear cells (CBMC) were obtained by Ficoll‐Hypaque gradient centrifugation, and analysed by flow cytometry. Fixable viability dye Efluor780 (FVD) was used to discriminate dead cells. The following anti‐human fluorescence conjugated antibodies and their corresponding isotype controls used were as follows: anti‐human CD4‐APC (clone OKT4, Biolegend), FVD (BD), anti‐human CXCR5‐PE (clone J252D4, Biolegend), anti‐human PD‐1‐FITC (clone A17188B, Biolegend) and anti‐human CD19‐Percpcy5.5 (clone 4G7, Biolegend). Cells were washed with cold PBS for three times, and resuspended in fluorescence activating cell sorter flow buffer (PBS containing 0.1% BSA and 0.04% EDTA‐Na_2_) to a density of 1 × 10^6^ cells/mL and then incubated for 15 minutes at room temperature in the dark place with 2 μL fluorochrome‐conjugated Abs. Tfh cells were defined as CD4^+^CXCR5^+^PD‐1^+^ and B cells as CD19^+^. Data were analysed with FlowJo 10.0 software package (Treestar Inc).

### 2.3 Magnetic beads separation

Naïve CD4^+^ T Cell Isolation Kit II (Miltenyi Biotec) was used to isolate naïve CD4^+^ T cells from CBMC. Briefly, determined cell number and viability (>99%) by Trypan blue staining. Then, resuspend cell pellet in 40 μL of buffer, added 10 μL of biotin‐antibody cocktail per 10^7^ total cells and incubated for 5 minutes in 4°C. Added 20 μL of buffer and 20 μL of anti‐biotin microbeads per 10^7^ total cells. Added 10 μL of CD44 microbeads per 10^7^ total cells and incubated for 10 minutes in 4°C. Finally, naïve CD4^+^ T cells were sorted (purity >95%, viability >99%) by column in the magnetic field of a suitable magnetic activating cell separator.

### 2.4 Cell culture

Naïve CD4^+^ T cells were suspended in RPMI 1640 medium (Gibco) supplemented with 2 mM L‐glutamine, 10 mM HEPES, 20 mM 2‐methoxyestradiol, 150 U/mL streptomycin, 200 U/mL penicillin and 10% foetal bovine serum. Then, naïve CD4^+^ T cells (2 × 10^5^) were transferred into 96‐well plates, which were plate bound with 5 μg/mL anti‐human CD3 purified antibody (OKT3, eBioscience) overnight in 4°C conditions. Treated with 1 μg/mL anti‐human CD28 purified antibody (CD28.2, eBioscience), 5 ng/mL Recombinant Human TGF‐β1 Protein (R&D), 10 ng/mL Recombinant Human IL‐23 Protein (R&D), 10 ng/mL Recombinant Human IL‐6 (R&D), 0‐20 μM E_2_ (Sigma‐Aldrich). The cultures were maintained at 37°C in a 5% CO_2_‐humidified atmosphere. Medium was changed on the third day. The proportion of CD4^+^CXCR5^+^PD‐1^+^ Tfh cells were analysed by flow cytometry on the sixth day.

### 2.5 Enzyme‐linked immunosorbent assays (ELISA)

Serum samples collected from patients were kept at −80°C. Enzyme immunoassays were performed to determine the concentration of IL‐21 and IL‐6 using ELISA kits (Biolegend). Human serum was diluted 1:1 in sample buffer and incubated for 30 minutes. Following three washes with the washing buffer, the bound antibodies were detected by incubation with anti‐human IgG HRP conjugate for 30 minutes. Then, the samples were washed again as described earlier, and tetramethyl benzidine substrate was added for 15 minutes. The optical density was read at 450 nm using an automated spectrophotometer. All samples were assayed in duplicate.

### 2.6 Quantitative real‐time polymerase chain reaction (qRT‐PCR)

Initially, total RNA was extracted from PBMC using AxyPrep Multisource Total RNA Miniprep Kit (Axygen). The RNA quality was checked based on the A_260_/A_280_ ratio, and pure RNA samples were converted to cDNA using PrimeScript RT Reagent Kit with gDNA Eraser in accordance with the manufacturer's instructions (Takara Bio). The polymerase chain reaction was performed using SYBR Green based on the instruction manual (Takara Bio). The primer sequences are presented in Table 1 (Comate Bioscience). The relative mRNA expression of different genes was quantified using the ∆∆C*_t_* method. *Β‐actin* was used as a housekeeping gene.

**TABLE 1:** Oligonucleotide and primer sequence

Oligonucleotide	Primer sequence
β‐actin	5′‐TTCAACACCCCAGCCATG‐3′ (forward) 5′‐CCTCGTAGATGGGCACAGT‐3′ (reverse)
BCL‐6	5′‐GGAGTCGAGACATCTTGACTGA‐3′ (forward) 5′‐ATGAGGACCGTTTTATGGGCT‐3′ (reverse)
BACH2	5′‐AATACCAGCTTGCATGTACCAA‐3′ (forward) 5′‐TTATCTTCCCGGAATGTGCTTG‐3′ (reverse)
GPER1	5′‐ CACCAGCAGTACGTGATCGG ‐3′ (forward) 5′‐ CATCTTCTCGCGGAAGCTGAT ‐3′ (reverse)
XBP1	5′‐ CCCTCCAGAACATCTCCCCAT ‐3′ (forward) 5′‐ ACATGACTGGGTCCAAGTTGT ‐3′ (reverse)
IRF4	5′‐ GCTGATCGACCAGATCGACAG ‐3′ (forward) 5′‐ CGGTTGTAGTCCTGCTTGC ‐3′ (reverse)

### 2.7 Hormone assays

Serum E_2_ was measured by electro‐chemiluminescence immunoassay (ECLIA, Cobas Roche Diagnostics). Intra‐ and inter‐assay variations were less than 8% and 11%, respectively.

### 2.8 Network construction, and gene oncology (GO) analysis, pathway analysis

String (https://string‐db.org/), a powerful tool aiming to construct a comprehensive and objective global network, it contains direct (physical) as well as indirect (functional) interactions.^30^ GeneMANIA (http://genemania.org/) is a user‐friendly web interface used for analysing gene functions, generating gene lists and prioritizing genes for functional assays.^31^ After selecting *homo sapiens* from the nine optional organisms, and entering the genes of interest into the search bar, the results showed. Potential targets were uploaded to the Metascape server (http://metascape.org/) for GO analysis, which was used to identify functionally related genes from the list of differentially expressed genes. Functionally related genes were usually categorized to biological processes, cellular components and molecular functions. A false discovery rate<5.0 and *P* value <.05 were considered statistically significant.

### 2.9 Statistical analysis

GraphPad Prism version 7.00 and SPSS version 21.0 software were used for statistical analysis. All data were presented as the mean ± SD. Variables between groups were analysed using the independent‐samples Student's *t* test or chi‐squared tests, and *P* < .05 was considered statistically significant.

## 3 RESULTS

### 3.1 The proportions of Tfh and B cells in PBMCs was positively related to the serum E_2_ level

**FIGURE 1:**
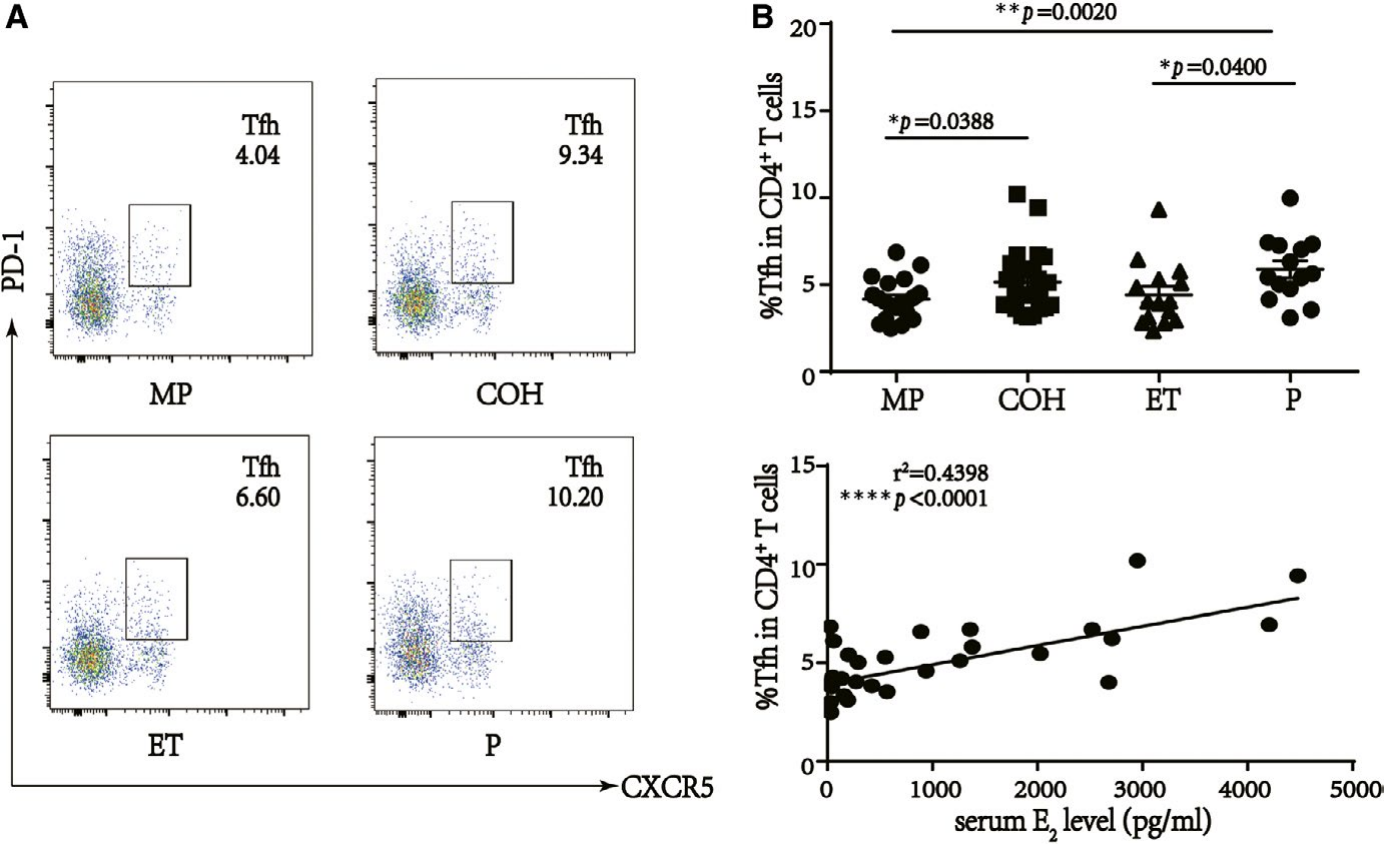
Percentages of T follicular helper (Tfh) cells are positive related to serum estradiol (E_2_) level in in vitro fertilization (IVF) patients. A, Percentages of Tfh cells in live CD4^+^ T cells. B, Percentages of Tfh cells are higher in controlled ovarian hyperstimulation (COH) and pregnant after embryo transfer (P) groups than menstrual period (MP) group (*P = .0388 and **P = .0020, respectively). No difference between the COH and P groups (*P* = .2342). C, Positive correlation between the percentages of Tfh cells in PBMCs and serum E_2_ level in MP and COH groups (*r* ^2^ = .4398, ****P < .0001, n = 39)

To investigate the correlation between the proportions of Tfh and B cells, and the serum E_2_ level in human beings, we first analysed these parameters in the blood of women who underwent IVF treatment. Tfh cells were identified as CD4^+^CXCR5^+^PD‐1^+^, whereas B cells were defined as CD19^+^. As expected, serum E_2_ levels were higher in COH, ET, P groups compared with MP group (Supplemental Figure [Link], [Link]; *P* = .0009, *P* = .0039 and <.0001, respectively). Meanwhile, as shown in Figure 1A,B, the percentage of CD4^+^ T cells was significantly elevated in the COH and P groups compared with that in the MP group (*P* = .0388 and *P* = .0020, respectively). We also investigated the correlation between Tfh cell percentages and serum E_2_ levels in the MP and COH groups without progesterone interference, the result showed a positive relationship (Figure 1C; *r* ^2^ = .4398, *p* < .0001, n = 39). Relationship between B cell percentages and serum E_2_ levels showed a linear relationship (Supplemental Figure [Link], [Link]; *r* ^2^ = .1306, *P* = .0147, n = 39).

**FIGURE 2:**
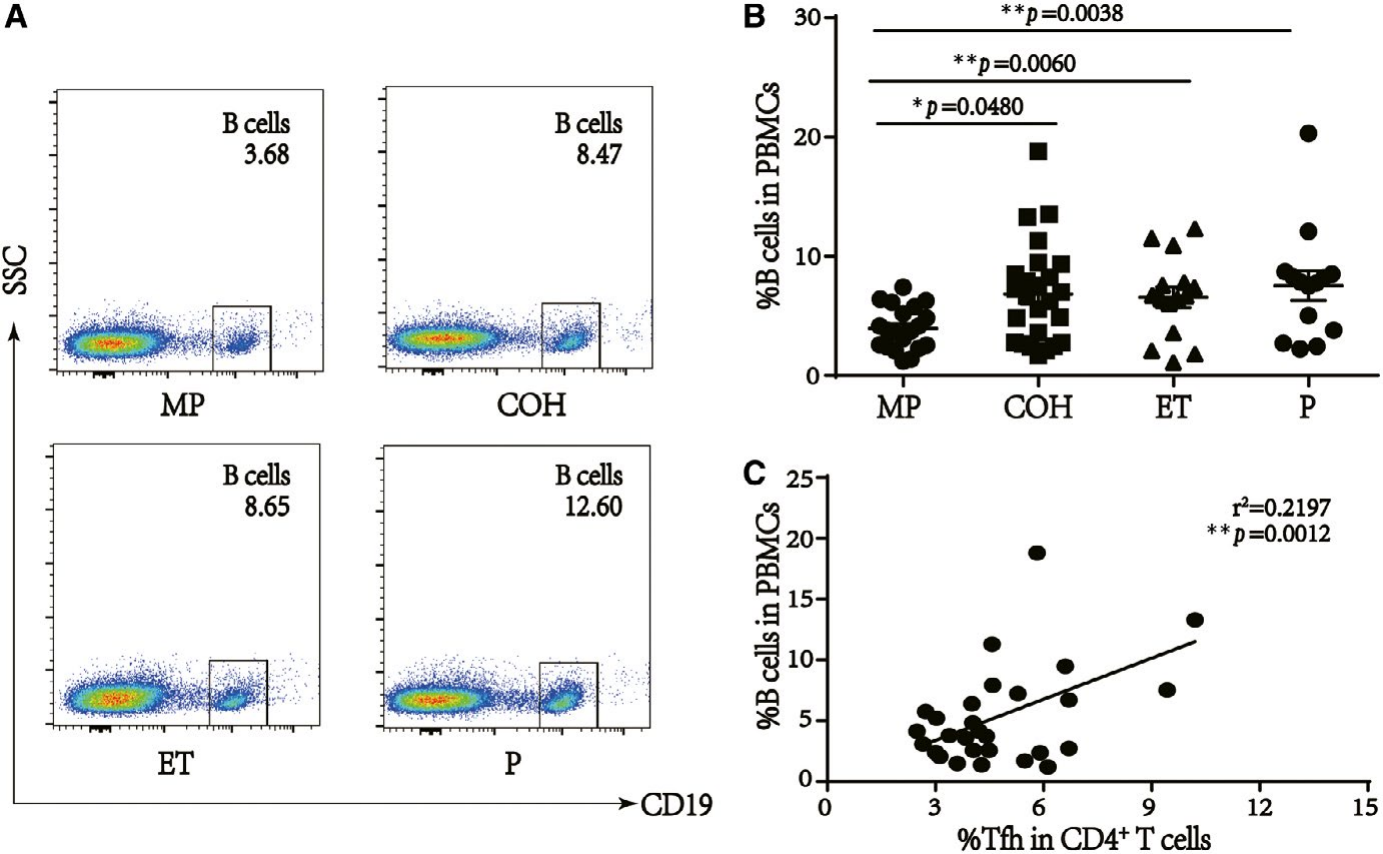
Percentages of B cells are positive related to Tfh cells in IVF patients. A, Percentages of B cells in live cells. B, Percentages of B cells are higher in COH, embryo transfer (ET) and P groups than MP group (*P = .0480, **P = .0060 and **P = .0038, respectively). C, Positive correlation between the percentage of B cells in PBMCs and serum E_2_ level in MP and COH groups (*r* ^2^ = .2197, **P < .0012, n = 39)

As Tfh cells play crucial roles in the differentiation and maturation of B cells,^13^ we next examined changes in the B cell percentage in PBMCs. We found that the percentage of B cells was significantly increased in COH, ET and P groups compared with that in MP group (*P* = .0480, *P* = .0060 and *P* = .0038, respectively) (Figure 2A,B). Furthermore, the B cell percentage in PBMCs was positively correlated with the percentage of Tfh cells among CD4^+^ T cells (Figure 2C; *r* ^2^ = .2197, *P* = .0012, n = 39).

### 3.2 E_2_ promoted Tfh differentiation and enhanced plasma cell production

**FIGURE 3:**
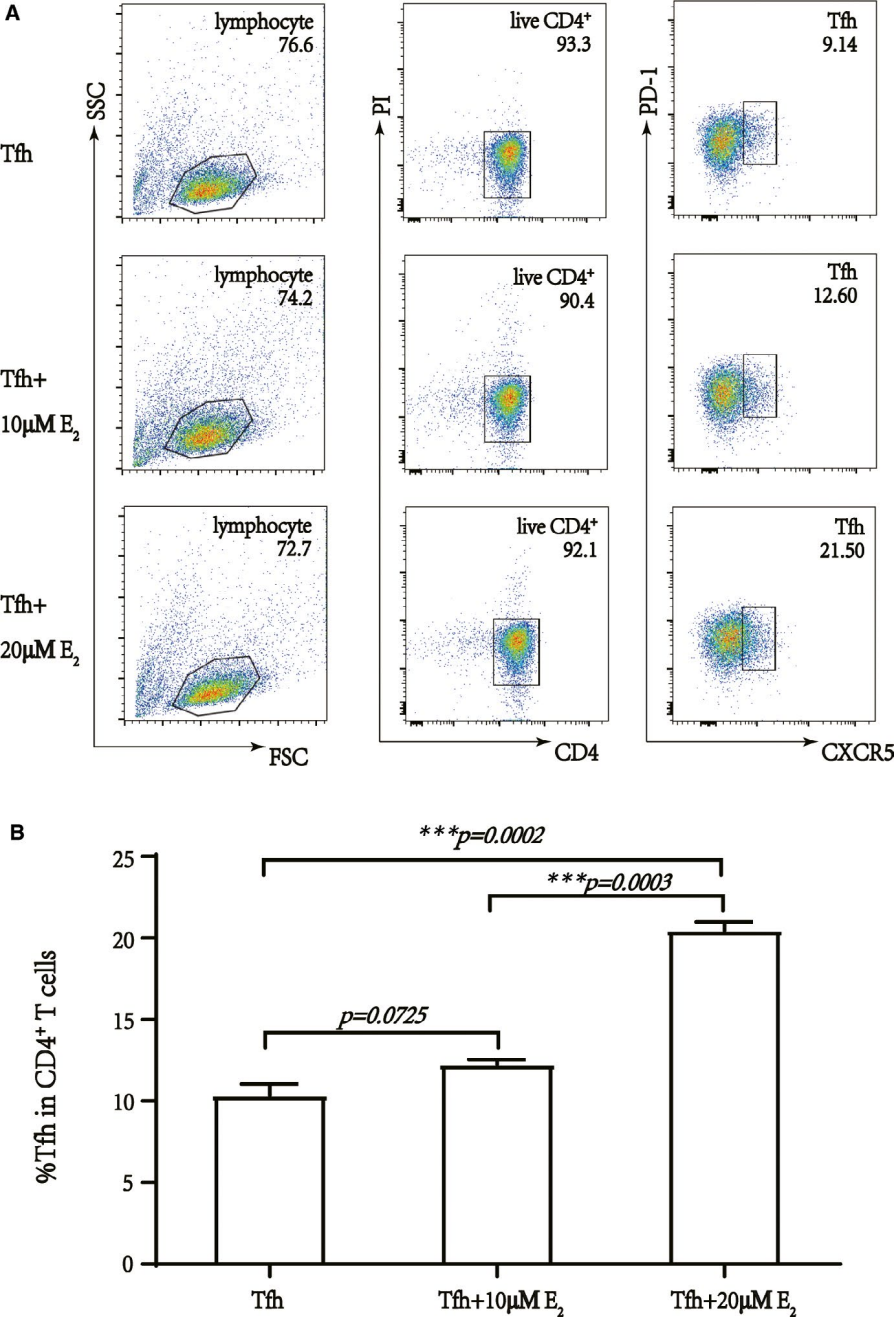
17β‐Estradiol induces Tfh cells differentiation in vitro. A, The flow cytometry analysis showed that 17β‐estradiol‐induced Tfh cells in a concentration‐dependent manner. B, Qualification of Tfh cells in total CBMC

To clarify whether E_2_ increased the percentage of Tfh cells by enhancing their differentiation, we separated naïve CD4^+^ T cells from CBMCs and treated them with various concentrations of E_2_ under CD3/CD28 stimulation and conditions suitable for Tfh cell differentiation (TGF‐β1, IL‐23, IL‐6 co‐culture) in vitro. After a 6‐day culture, flow cytometry confirmed that the percentage of Tfh cells was significantly augmented after treatment with 20 µM E_2_ (Figure 3).

**FIGURE 4:**
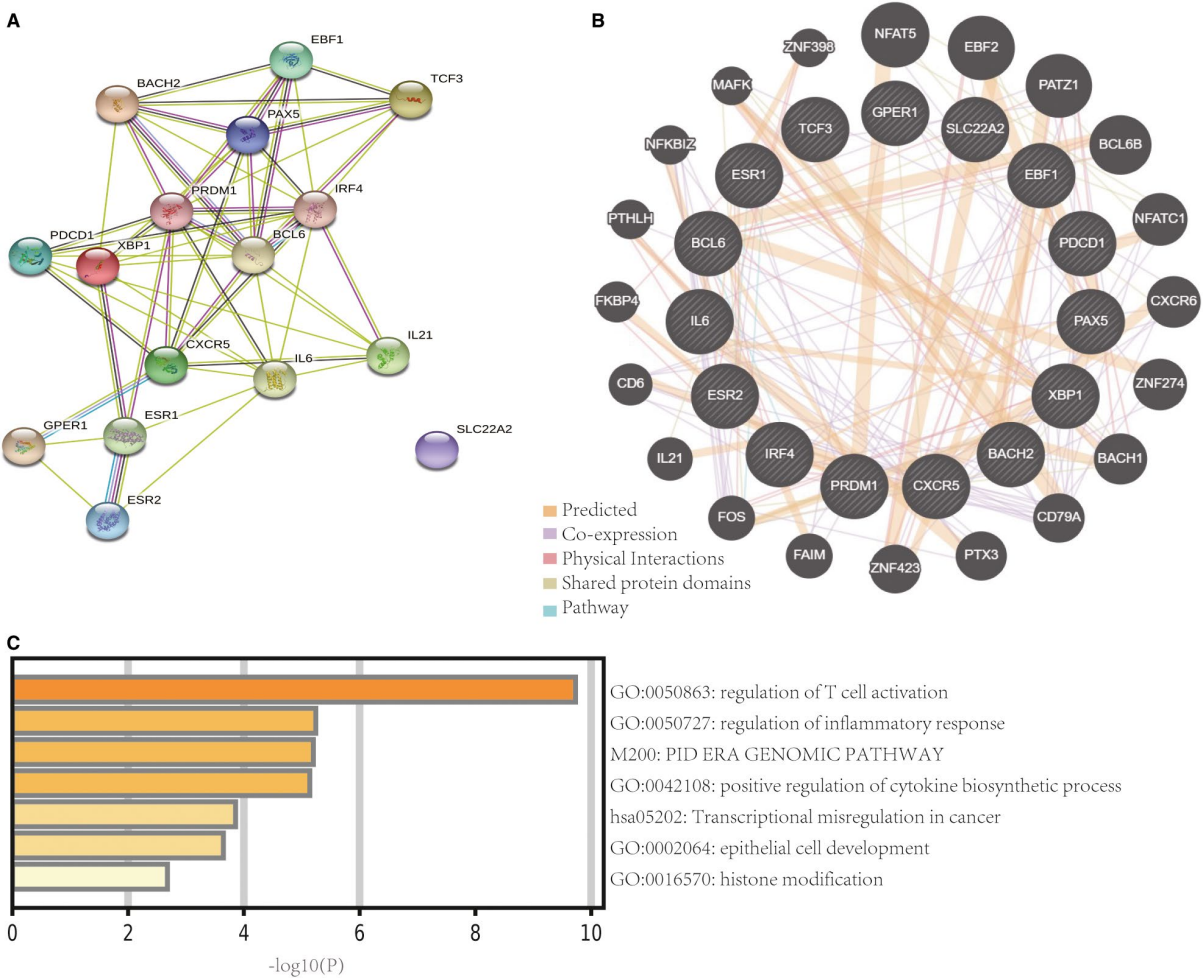
Bioinformatic analysis of Tfh and B cell biomarkers. A, Protein‐protein interaction networks of cytokines, and Tfh and B cell transcription factors. B, Biomarker networks of Tfh and B cells. Black protein nodes indicate target proteins. Different connecting colours represent different correlations. The functional association of targets was analysed using GeneMANIA. Genes in black circles were submitted as query terms in searches. Grey circles indicate genes associated with query genes. C, Gene Ontology analysis of targets. The *y*‐axis shows significantly enriched Biological Process categories of the targets, and the *x*‐axis shows the enrichment scores of these terms (*P < .05)

Next, bioinformatics was used to clarify the potential mechanism underlying these interactions. We inputted BCL6, PD‐1, CXCR5, BACH2, PAX5, EBF, BLIMP1, E2A, OCT2, IRF4, XBP1, IL‐6, IL‐21, Erα, Erβ and G protein‐coupled oestrogen receptor (GPER) into STRING to establish protein‐protein interaction networks and perform a functional enrichment analysis (Figure 4A). GeneMANIA revealed interacting proteins that were functionally similar to these 16 genes; 49.8% were predicted, 40.8% had matching co‐expression characteristics and 6.13% displayed similar physical interactions (Figure 4B). To access probable signalling mechanisms, an analysis of the interaction networks using Metascape showed that 93.4% of the genes were enriched. The top three enriched pathways were regulation of T cell activation (GO:0050863), regulation of inflammatory response (GO:0050727), and positive regulation of the cytokine biosynthetic process (GO:0042108) (Figure 4C); these were dominantly enriched for GPER‐1, IL‐6, IL‐21, BCL6, CXCR5, PD‐1, IRF4 and XBP‐1.

### 3.3 Serum IL‐6 and IL‐21 levels correlated with the E_2_ level

**FIGURE 5:**
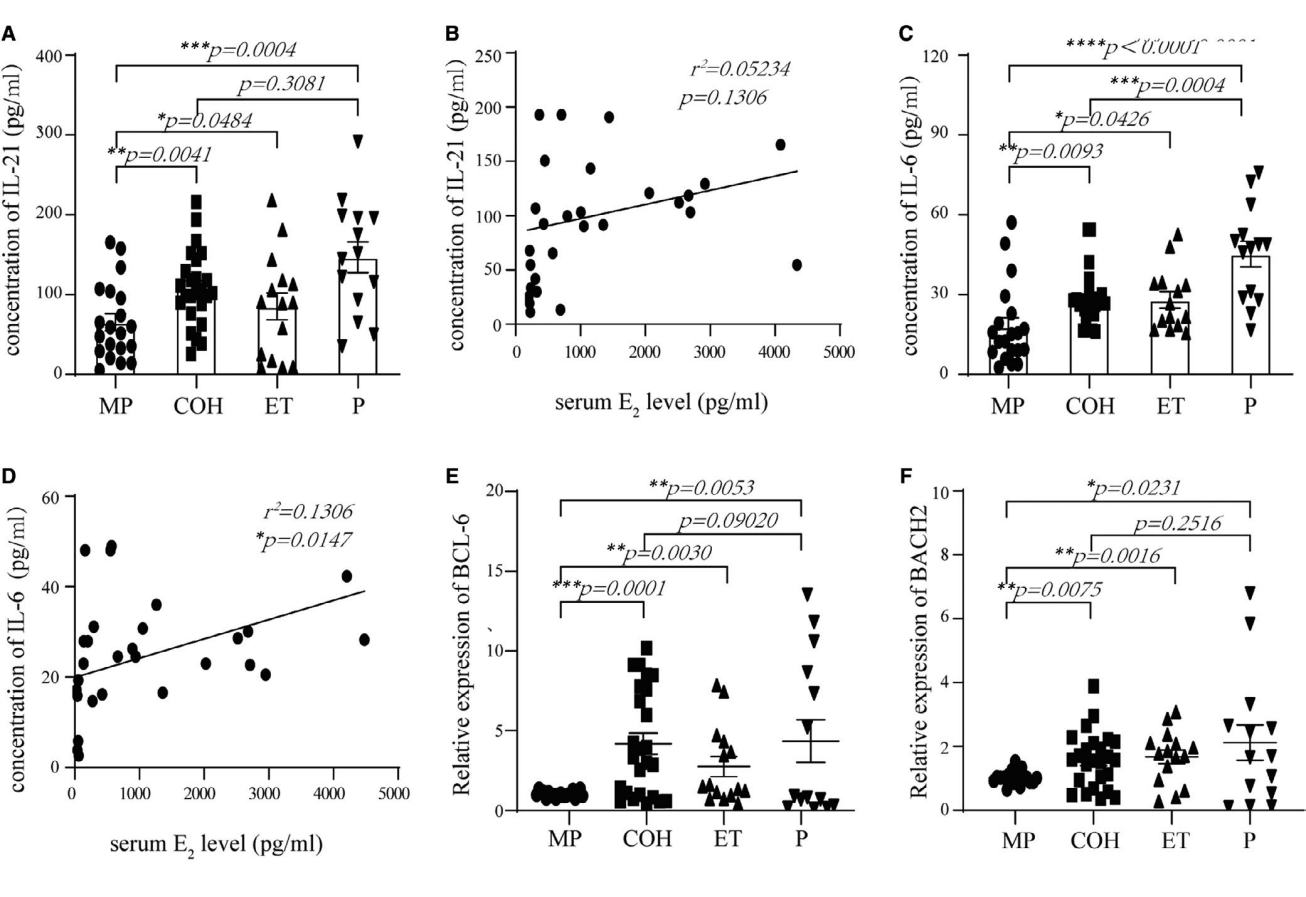
Serum IL‐21 / IL‐6 levels, and relative expression of BCL‐6 / BACH2 in PBMC are higher in COH, ET and P groups than MP group. Serum IL‐21 and IL‐6 levels are positive correlated with serum E2 level. A, Serum IL‐21 levels is higher in COH, ET and P groups than MP group (**P = .0041, *P = .0484, and ***P = .0004, respectively). B, Positive correlation between serum IL‐21 and E_2_ levels in MP and COH groups (*r* ^2^ = .05234, *P* = .1306, n = 45). C, Serum IL‐6 levels is higher in COH, ET and P groups than MP group (**P = .0093, *P = .0426, and****p < .0001, respectively). D, Positive correlation between serum IL‐6 and E_2_ levels in MP and COH groups (*r* ^2^ = .1306, *P = .0147, n = 45). E, Relative expression of BCL‐6 in PBMCs is higher in COH, ET and P groups than MP group (***P = .0001, **P = .0030, and **P = .0053, respectively). F, Relative expression of BACH2 in PBMCs is higher in COH, ET and P groups than MP group (**P = .0075, **P = .0016, and *P = .0231, respectively)

IL‐21 and IL‐6 are not only secreted by Tfh cells, but also promote Tfh cell differentiation.^32^ Therefore, we measured the levels of IL‐21 and IL‐6 in serum using ELISAs (Figure 5A,C) and analysed the correlation between IL‐21, IL‐6 and E_2_ levels (Figure 5B,D). Our data demonstrated that under endogenous E_2_ treatment alone (the MP and COH groups, n = 39), without progesterone interference, both IL‐21 and IL‐6 levels were up‐regulated significantly in serum (*P* = .0041 and *P* = .0093, respectively); however, IL‐21 levels had no linear correlation with E_2_ levels.

### 3.4 E_2_ increased Tfh and B cell transcription factor expression

**FIGURE 6:**
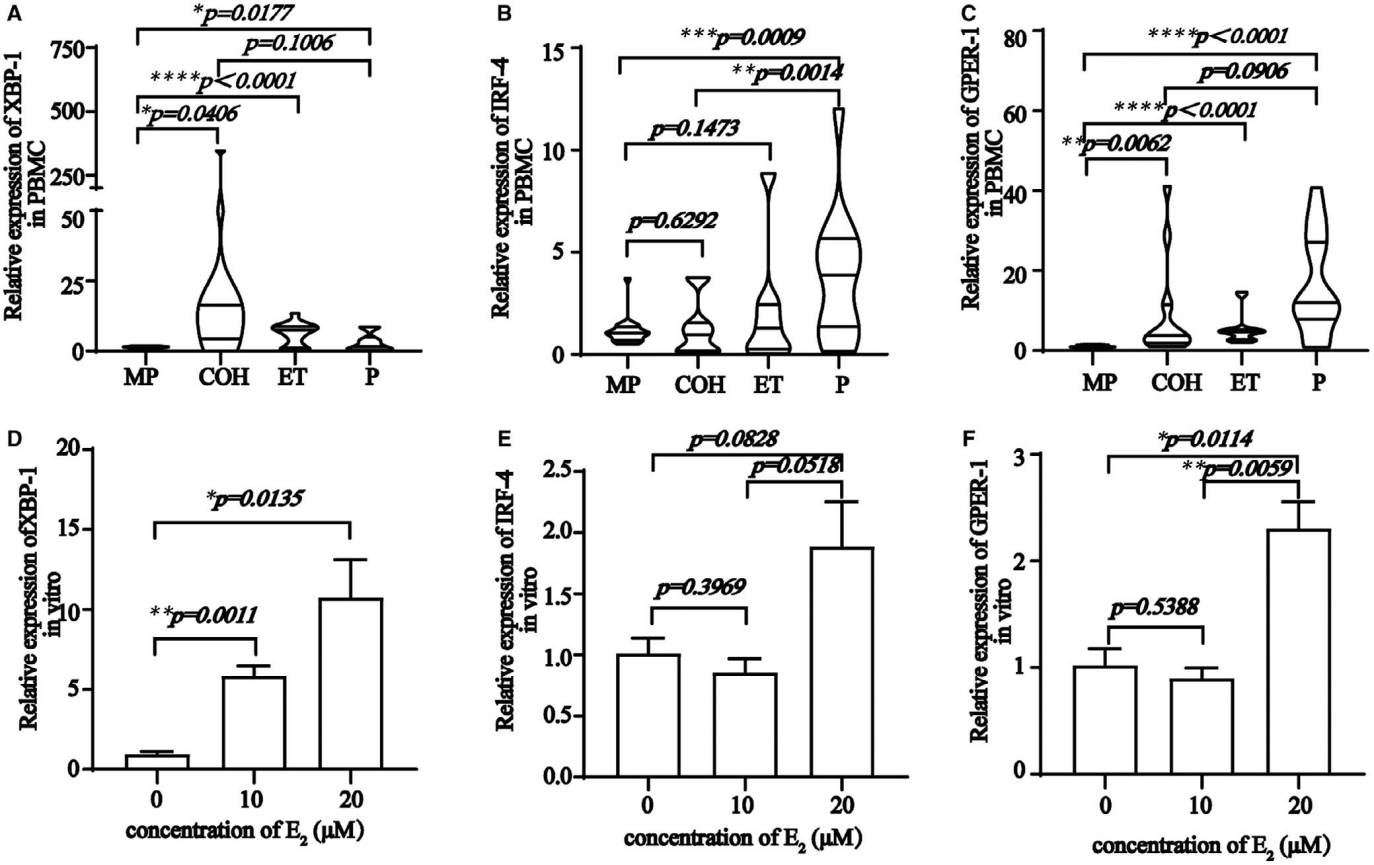
Relative expression of XBP‐1, IRF‐4, and GPER‐1 in PBMC are higher in COH, ET and P groups than MP group. Relative expression of XBP‐1, IRF‐4, and GPER‐1 in Tfh cells differentiation system are higher with 17β‐Estradiol. A, Relative expression of XBP‐1 in PBMCs is higher in COH, ET and P groups than MP group (*P = .0406, ****P < .0001, and *P = .0177, respectively). B, Relative expression of IRF‐4 in PBMCs is higher in P groups than MP group (***P = .0009). C, Relative expression of GPER‐1 in PBMCs is higher in COH, ET and P groups than MP group (**P = .0062, ****P < .0001, and ****P < .0001, respectively). D, Relative expression of XBP‐1 in Tfh cells differentiation condition in vitro with various E_2_ concentrations (0 vs 10 μM, **P =.0011; 0 vs 20 μM, *P =.0135). E, Relative expression of IRF‐4 in Tfh cells differentiation condition in vitro with various E_2_ concentrations (0 vs 10 μM, *P* = .3969; 0 vs 20 μM, *P* = .0828; 10 vs 20 μM, *P* = .0518). F, Relative expression of GPER‐1 in Tfh cells differentiation condition in vitro with various E_2_ concentration (0 vs 10 μM, *P* = .5388; 0 vs 20 μM, *P = .0114; 10 vs 20 μM, **P = .0059)

Besides IL‐6, IL‐21 and BCL6 (known markers of Tfh cells), recent studies identified BACH2 as a key transcription factor in antibody class switching.^33^, ^34^ The function of BACH2 in Tfh cells is still controversial. Thus, we detected its expression and that of BCL6 and BACH2 i*n vivo* (Figure 5E,F). The expression of BACH2 was in accordance with that of IL‐21 and IL‐6. As a crucial transcription factor of plasma cells, the expression of *XBP1* increased consistently with E_2_ levels, which further verified the relationship between E_2_ and B cells. Meanwhile, expression of *IRF4* and *GPER1* were consistent with the bioinformatics analysis, both in vivo and in vitro, indicating that *IRF4* and *GPER1* expression in Tfh and B cells were crucial for their augmentation (Figure 6).

## 4 DISCUSSION

In this study, we revealed a positive correlation between the numbers of Tfh and B cells, and E_2_ levels, during IVF. Combined with in vitro Tfh differentiation and a bioinformatics analysis, we speculated that E_2_ may affect Tfh cell numbers and favour humoural immunity through GPER1 and transcription factors, including BCL6, BACH2, XBP1 and IRF4. This indicated that the regulation of Tfh cells might be a novel target to modulate the maternal‐foetal immune balance.

E_2_ generally works through its receptor, which is widely expressed on the surface and intracellular of various cells. High E_2_ levels in the serum may indicate enhanced pro‐inflammatory T cells, such as Th1 and Th17 subsets, instead of protective Th2 and T regulatory subgroups.^4^, ^5^ Oestrogen receptors (ERs) can be divided into three major subsets: nuclear receptor ERα, ERβ, and GPER.^35^, ^36^ *BCL6, PD‐1, CXCR5* and *BACH2* are functional genes for Tfh cells, while IL‐6 and IL‐21 are the released cytokines. PAX5, EBF, BLIMP1, E2A, OCT2, IRF4 and XBP1 might be crucial transcription factors involved in different stages of B cell differentiation.^37^ Meanwhile, E_2_ plays a dichotomous role in augmenting myeloid‐derived suppressor cells while inhibiting T cell functions.^38^ Furthermore, IL‐6 is commonly secreted by different subgroups of activated T cells, as well as others such as myeloid‐derived suppressor cells.^38^ Our data demonstrated that under endogenous E_2_ treatment alone, without progesterone interference, both IL‐21 and IL‐6 levels were up‐regulated significantly in serum; however, IL‐21 levels had no linear correlation with E_2_ levels. This finding might be because, during pregnancy and supraphysiological E_2_ stimulation, cell subsets change and the complex microenvironment might contribute to higher IL‐6 concentrations.

Here, we proposed that E_2_ might play a dominant role in Tfh cell recruitment and differentiation. We defined Tfh cells as CD4^+^CXCR5^+^PD‐1^+^. It is well‐established that IL‐6 is a potent inducer of IL‐21, which has been observed in activated murine CD4^+^ T cells.^13^ IL‐6 also drives CXCR5 expression ^16^ and induces transient BCL6 expression.^32^ IL‐6 deficiency results in the severe reduction of CXCR5^+^BCL6^+^ early Tfh cells in vivo within 72 hours of an acute viral infection.^39^ In some diseases, PD‐1^+^ Tfh cells are considered better B cell activators than the PD‐1^‐^ subset.^40^

Both the Tfh and B cell percentages were elevated in the COH group, with no significant difference compared with those in the P group. Although the percentages of Tfh and B cells in the ET group were notably higher than those in the MP group, they were slightly lower than those in the COH group. This indicates that E_2_ might play a crucial role in augmenting Tfh and B cells during pregnancy, independent of progesterone. Furthermore, after inducing Tfh cells in vitro, they exhibited enhanced differentiation upon E_2_ treatment, as expected.

BACH2, another important transcription factor to maintain the phenotype of GC B cells,^41^ is also expressed by T cells and directs their differentiation, homeostasis and effector functions.^34^, ^42^ *BACH2* is highly expressed in GC B cells and is required for antibody class switching and blocking plasma cell differentiation.^43^ In addition, BACH2‐mediated regulation can suppress the CXCR5 promoter via the site 1 negative regulatory element during Tfh cell differentiation.^44^ Strikingly, in contrast to GC B cells, Tfh cells are characterized by low expression of BACH2 in mice. *BACH2* overexpression in Tfh cells inhibits a specific set of genes, including *IL‐21* and *BCL6,* indicating that the function of this transcription factor is reversed in T cells.^45^ Previous studies suggested that *BACH2* is associated with numerous autoimmune and allergic diseases in humans by inducing excessive pathogenic Tfh cells,^46^ which is opposite to results observed in mice. Little is known about the contrasting effects of BACH2 in mice and humans; therefore, further research is needed.

Both IRF4 and XBP1 play important roles in plasma B cell differentiation, immunoglobulin secretion and isotype switching.^47^, ^48^ IRF4 has been confirmed as a crucial transcription factor in Tfh cell differentiation.^49^ Our bioinformatics analysis showed that the ER, Tfh and B cell transcription factors, IRF4 and XBP1, were enriched. Hence, our results demonstrated that, upon E_2_ exposure, a forward loop was formed in which GPER1 was activated to increase *BCL6, BACH2, XBP1* and *IRF4* expression, thereby driving Tfh and plasma cell augmentation.

Numerous mouse studies have supported the vital role of Tfh cells in autoimmune diseases.^50^, ^51^, ^52^ In addition, increased E_2_ and Tfh cell levels have been observed frequently in human autoimmune diseases, such as systemic lupus erythematosus, and are positively correlated with serum autoantibody titres and disease severity.^53^, ^54^ This suggests an important role of E_2_ and Tfh cells in the pathogenesis of these diseases.

## 5 CONCLUSIONS

We demonstrated that Tfh cells, as a novel subset of immune cells involved in immune tolerance at the maternal‐foetal interface, are influenced by circulating E_2_ levels. Tfh cells also regulate cytokines and transcription factors, contributing to enhanced humoural immunity. Further investigation of these important results will advance our understanding of the clinical application of Tfh cells as a therapeutic target for immune‐related miscarriage and IVF failure.

## 6 ETHICS STATEMENT

The Ethics Review Boards of the First Hospital of Jilin University (No. 2018‐069) and the Jilin Province People's Hospital (No.2018‐Y‐005) approved this research. Study participants and/or their legal guardians provided written informed consent.

## CONFLICT OF INTEREST

The authors declare no conflicts of interest.

## AUTHOR CONTRIBUTION

**Cong Hu:** Data curation (lead); Formal analysis (lead); Writing‐original draft (lead). **Honglei Liu:** Data curation (equal); Formal analysis (equal); Writing‐original draft (equal). **Bo Pang:** Data curation (equal); Methodology (equal); Software (lead); Visualization (lead). **Hao Wu:** Investigation (equal); Resources (equal); Visualization (equal). **Xiuying Lin:** Resources (lead). **Yu Zhen:** Conceptualization (equal); Project administration (equal); Resources (equal). **Huanfa Yi:** Conceptualization (lead); Project administration (lead); Supervision (lead); Writing‐review & editing (lead).

## Supporting information

FigS1‐Legends

FigS1

TableS1

## Data Availability

The data that support the findings of this study are available from the corresponding author upon reasonable request.
