# Supplementary material for: Supraphysiological estradiol promotes human T follicular helper cell differentiation and favours humoural immunity during in vitro fertilization
Source: J Cell Mol Med. 2021 May 24;25(14):6524–34. doi: 10.1111/jcmm.16651 (PMC8278094; doi:10.1111/jcmm.16651)
Supplement: Supplementary file 1 — FigS1‐Legends [file JCMM-25-6524-s001.docx]

**Supplemental Figure 1.** **Serum E_2_ level in IVF patients, and statistical analysis of the correlation between percentages of B cells in PBMCs and serum E_2_ level.** (A) Statistical analysis of the four groups. COH, ET and P groups showed significantly difference with MP, COH ****p*=0.0009, ***p*=0.0039 and *****p*＜0.0001, respectively. (B) There is a correlation between the percentages of B cells in PBMCs and serum E_2_ level in MP and COH groups (*r*^2^ = 0.1306, **p*=0.0147, n = 39).
