# Supplementary figures and images for: Supraphysiological estradiol promotes human T follicular helper cell differentiation and favours humoural immunity during in vitro fertilization
Source: J Cell Mol Med. 2021 May 24;25(14):6524–34. doi: 10.1111/jcmm.16651 (PMC8278094; doi:10.1111/jcmm.16651)

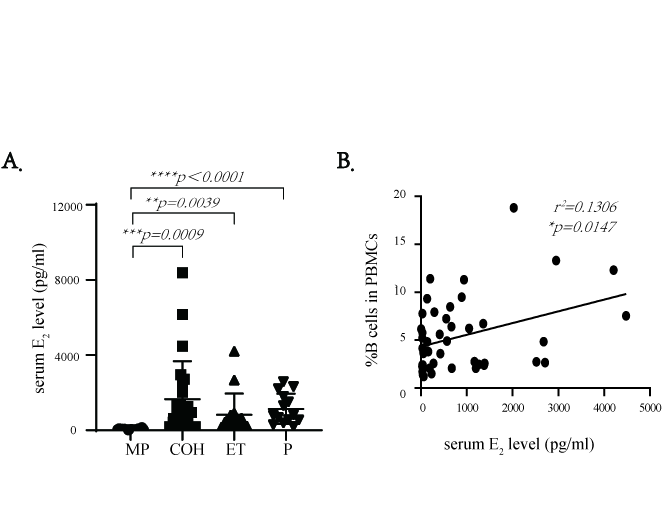

Supplement: Supplementary file 2 — FigS1 [file JCMM-25-6524-s003.tif]
