# Supplementary material for: Supraphysiological estradiol promotes human T follicular helper cell differentiation and favours humoural immunity during in vitro fertilization
Source: J Cell Mol Med. 2021 May 24;25(14):6524–34. doi: 10.1111/jcmm.16651 (PMC8278094; doi:10.1111/jcmm.16651)
Supplement: Supplementary file 3 — TableS1 [file JCMM-25-6524-s002.docx]

**Supplementary Table 1 Patient characters**

|  | **MP** | **COH** | **ET** | **P** |
| --- | --- | --- | --- | --- |
| **Count** | 20 | 25 | 15 | 14 |
| **Mean Age (year)** | 31.29 | 30.96 | 32.29 | 29.86 |
| **SD Age (year)** | 4.97 | 4.65 | 4.73 | 3.25 |
| **Median Age (year)** | 30 | 30 | 31 | 29.5 |
| **Min Age (year)** | 35 | 35 | 35 | 33 |
| **Max Age (year)** | 24 | 24 | 23 | 24 |

Note: There were no statistically significant differences among the groups. SD, standard deviation; Min, minimum; Max, maximum.
